# Emotional comorbidities in epilepsy result from seizure-induced corticosterone activity

**DOI:** 10.1016/j.ynstr.2024.100678

**Published:** 2024-10-11

**Authors:** Renaud C. Gom, Antis G. George, Sydney A. Harris, Pasindu Wickramarachchi, Dhyey Bhatt, Shaona Acharjee, Quentin J. Pittman, Matthew N. Hill, Roberto Colangeli, G. Campbell Teskey

**Affiliations:** a Hotchkiss Brain Institute, Cumming School of Medicine, University of Calgary, Calgary, Alberta, T2N 4N1, Canada; b Department of Cell Biology and Anatomy, University of Calgary, Calgary, Alberta, T2N 4N1, Canada; c Department of Physiology and Pharmacology, University of Calgary, Calgary, Alberta, T2N 4N1, Canada; d Mathison Centre for Mental Health Research and Education, University of Calgary, Calgary, Alberta, T2N 4N1, Canada; e Department of Experimental and Clinical Medicine, Università Politecnica Delle Marche, 60126, Ancona, Italy

**Keywords:** Epilepsy, Seizure, Stress, Endocannabinoids, Corticosterone, Emotional behaviour

## Abstract

People with epilepsy often have psychiatric comorbidities that can significantly impair their quality of life. We previously reported that repeated seizure activity persistently alters endocannabinoid (eCB) signaling in the amygdala which accounts for comorbid emotional dysregulation in rats, however, the mechanism by which these alterations in eCB signaling within the epileptic brain occur is unclear. Endocannabinoid signaling is influenced by corticosterone (CORT) to modulate cognitive and emotional processes and a hyperactive hypothalamic-pituitary-adrenal (HPA) axis occurs in both people with epilepsy and nonhuman animal models of epilepsy.

We employed selective pharmacological tools and a variety of approaches including whole-cell patch-clamp electrophysiology, behavioural paradigms and biochemical assays in amygdala kindled adult male Long-Evans rats. We aimed to determine whether seizures induce hypersecretion of CORT and the role this plays in eCB system dysregulation, impaired fear memory, and anxiety-like behaviours associated with seizure activity.

Plasma CORT levels were significantly and consistently elevated following seizures over the course of kindling. Pre-seizure administration with the CORT synthesis inhibitor metyrapone prevented this seizure-induced CORT increase, prevented amygdala anandamide downregulation, and synaptic alteration induced by seizure activity. Moreover, treatment with metyrapone or combined glucocorticoid receptor (GR)/mineralocorticoid receptor (MR) antagonists prior to each elicited seizure were equally effective in preventing chronically altered anxiety-like behaviour and fear memory responses.

Inhibiting seizure-induced corticosterone synthesis, or directly blocking the effects of CORT at GR/MR prevents deleterious changes in emotional processing and could be a treatment option for emotional comorbidities in epilepsy.

## 1 Introduction

Temporal lobe epilepsy (TLE) is the most common form of adult focal epilepsy and is characterized by recurrent seizures arising from the temporal lobes (Engel, 1996; Janszky et al., 2005). Individuals with TLE often have a high incidence of behavioural comorbidities which include but are not limited to anxiety and depression (Engel, 1996; Kanner, 2016; Josephson and Jetté, 2017). Repeated seizure activity can lead to significant structural and physiological changes to temporal lobe structures that are associated with the development of some psychiatric disorders (Kleen et al., 2012).

The brain's endocannabinoid (eCB) neurotransmitters anandamide (AEA) and 2-arachidonoylglycerol (2-AG) are actively recruited during seizures to modulate acute hyperexcitability by reducing neurotransmitter release at pre-synaptic terminals (Marsicano et al., 2003; Wallace et al., 2003; Katona and Freund, 2008; Colangeli et al., 2017, 2019). Experimental evidence from animal models of seizures and epilepsy point to maladaptation of the eCB system, which includes the 2 transmitters, their receptors as well as synthesis and degradation enzymes, as a potential mechanism for seizure-induced changes in the physiology of emotionally related brain structures that are involved in seizure expression (Chen et al., 2003, 2007, 2016; Colangeli et al., 2017, 2020, 2022; Farrell et al., 2021). In particular, our laboratory has reported that, within the basolateral part of the amygdala (BLA), AEA signaling subserves a tonic inhibitory control over excitatory terminals which appears to be critical for maintaining functional emotional processing. Indeed, the induction of repetitive seizures from kindling results in a reduction of AEA, but not 2-AG, content within the amygdala which is paralleled by an increased excitatory drive in BLA and by emotional alterations (Colangeli et al., 2020). Moreover we report that pharmacologically enhancing the availability of AEA restores kindled seizure-induced both physiological and behavioural impairments (Colangeli et al., 2017, 2020). However, the mechanism underlying seizure induced BLA AEA signaling deficiency is unknown, creating a significant gap in our understanding of how seizures impact emotional behaviour.

Multiple studies have shown evidence of a hyperactive hypothalamic-pituitary-adrenal (HPA) axis and corresponding elevations in glucocorticoid levels in both rodent models and people with TLE (Herman et al., 2005; Maguire and Salpekar, 2013; O'Toole et al., 2014; Wulsin et al., 2016). It is well established that chronic exposure to elevated stress hormones can produce structural and functional changes within the brain that contribute to the development and maintenance of symptoms in major psychiatric disorders (McEwen, 2004; Lupien et al., 2009). The eCB system is known to be exquisitely sensitive to corticosterone (CORT) (Hill and McEwen, 2010), and chronic exposure to CORT has been repeatedly found to impair AEA signaling throughout many brain structures (Hill et al., 2010c; Bowles et al., 2012; Gray et al., 2016; Vecchiarelli et al., 2021) in a similar manner to what we have previously found following kindling (Rademacher et al., 2008; Hill et al., 2009, 2010b; Bowles et al., 2012).

Here we show for the first time that a seizure-induced CORT surge acting at glucocorticoid receptors (GR) and mineralocorticoid receptors (MR) results in decreased AEA and reorganization of endocannabinoid function that ultimately manifests as changes to emotional behaviour. We also provide evidence for a potential treatment option in people with epilepsy who have these behavioural comorbidities.

## 2 Methods and materials

### 2.1 Animals

Adult male and female pathogen free Long-Evans rats weighing between 275 and 325g were obtained from Charles River (Saint Constant, QC). We examined the effects of a corticosterone synthesis inhibitor in both males and females and found no significant differences between the sexes on the seizure-induced CORT response (unpaired *t*-test, vehicle effect: t_(15)_ = 0.27, p = 0.79; metyrapone effect: t_(15)_ = 0.76, p = 0.46), the chronic shift in AEA (unpaired *t*-test, vehicle effect: t_(9)_ = 0.48, p = 0.65; metyrapone effect: t_(10)_ = 0.29, p = 0.78), anxiety metrics of the EPM (two-way ANOVA, Distance sex effect: F_(1,72)_ = 0.58, p = 0.44; Open entries sex effect: F_(1,72)_ = 0.28, p = 0.60; Time open sex effect: F_(1,72)_ = 1.37, p = 0.25) or fear-related freezing responses measured post-conditioning (two-way RM ANOVA, conditioning sex effect: F_(1,356)_ = 0.01, p = 0.92; 24-h retention sex effect: F_(1,623)_ = 0.013, p = 0.91; 48-h retention sex effect: F_(1,267)_ = 0.58, p = 0.45 (data not shown). Subsequently this study used male rats only. Rats were individually housed in clear cages in a constant temperature-controlled room and were maintained on a 12/12-h light/dark cycle with lights on at 7:00 a.m. All handling and experiments were carried out in the light phase. All experiments and protocols were approved by the University of Calgary Health Sciences Animal Care Committee and in accordance with the Canadian Council for Animal Care guidelines. Experiments were designed to adhere to the 3 Rs of animal research: replacement, refinement, and reduction (AC20-0170).

### 2.2 Stereotaxic surgery

Bipolar depth electrodes were constructed from Teflon coated stainless steel wire tipped with gold plated male amphenol pins (A-M Systems). Rats were anesthetized and maintained on 1–2:100 isoflurane to oxygen for the surgical procedure. Once positioned within the stereotaxic apparatus (David Kopf Instruments), rats assigned to all groups (sham and seizure) were implanted with a chronic bipolar electrode into the right BLA at these coordinates relative to bregma (anteroposterior, −2.8 mm; mediolateral, −4.8 mm; dorsoventral, −8.0 mm). The electrode was affixed to the skull using 3 mounting screws, Metabond quick adhesive (C&B), and dental acrylic (Lang Dental). Rats were given subcutaneous (SQ) meloxicam (2 mg/kg) and recovered for one week before experimentation.

### 2.3 Drugs

All drugs or their vehicles used in the experiments were administered subcutaneously 30 min prior to every electrically elicited (kindled) seizure or sham stimulation. Metyrapone (50 mg/kg/ml; Caymen Chemicals; 14994, in 5% polyethylene glycol, 5% tween, 90% saline), a potent 11-beta-hydroxylase inhibitor was used to prevent CORT synthesis. To determine whether GRs contribute to the biochemical and behavioural findings illustrated in this study we administered the highly selective GR antagonist CORT113176 (Beaudry et al., 2014; Meyer et al., 2020, 2024) which has a Ki (50%) of 0.26 nM at GRs, Ki (20%) of 10 μM for MRs, and essentially no binding for progesterone receptors (30 mg/kg/ml; Corcept Therapeutics, in 5% DMSO, 95% coconut oil). To determine whether MRs contribute to the biochemical and behavioural findings illustrated in this study the MR antagonist spironolactone (Bell et al., 2007) which has a Ki (50%) of 2.3 nM at MR, 32.6 nM at GR, and 400 nM at progesterone receptors (40 mg/kg/ml; Spiro; Sigma Aldrich, in 5% DMSO, 95% propylene glycol). For specific experiments both the GR and MR antagonists were administered together. Doses were selected based on previous publications (Roozendaall et al., 1996; Liu et al., 2000; Benvenuti et al., 2021) and from dose-response experiments conducted within our laboratory.

### 2.4 Seizure elicitation (electrical kindling)

**Fig. 1:**
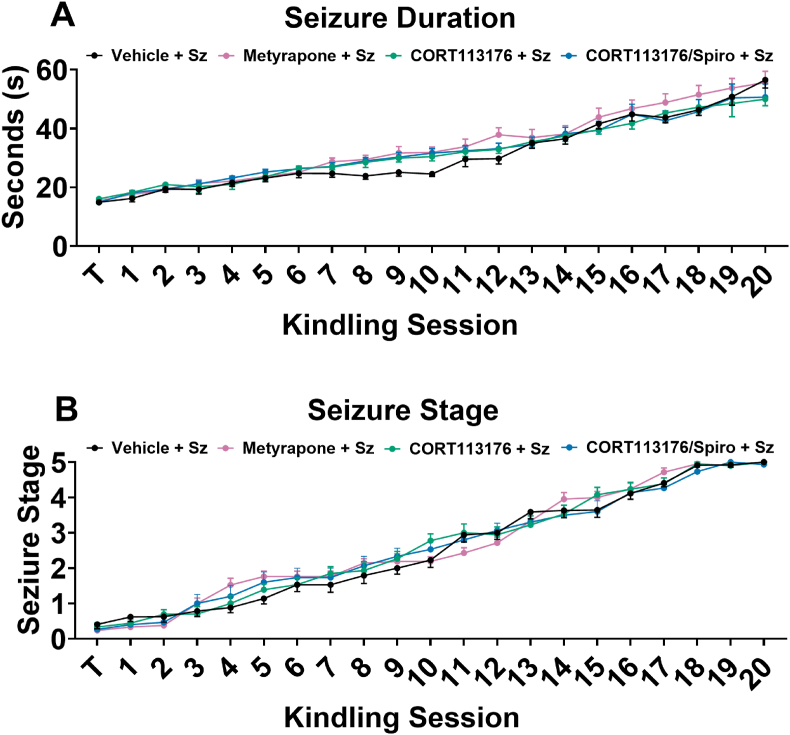
**Pre-seizure administration of metyrapone alone, CORT113176 alone, spironolactone alone, and CORT113176 with spironolactone did not alter seizure parameters**. Repeated electrical kindling of the BLA results in longer, more severe seizures over multiple sessions. **A)** There was no statistical difference in seizure duration at each session for rats administered vehicle alone, metyrapone alone, CORT113176 alone, spironolactone alone, or both CORT113176 and spironolactone (two-way RM ANOVA, day effect: F_(20,1776)_ = 131.60, p < 0.0001; drug effect: F_(4,1776)_ = 5.52, p = 0.0002; interaction: F_(80,1776)_ = 0.65, p = 0.99. **B)** There was no statistical difference in seizure stage at each session for rats administered vehicle alone, metyrapone alone, CORT113176 alone, spironolactone alone, or both CORT113176 and spironolactone (two-way RM ANOVA, day effect: F_(20,1776)_ = 377.20, p < 0.0001; drug effect: F_(4,1776)_ = 6.64, p < 0.0001; interaction: F_(80,1776)_ = 1.25, p = 0.007. “T” on each graph denotes the initial thresholding session. Data are expressed as means ± SEM. n = 15–22 per group.

Kindling is a form of sensitization where epileptiform discharges become progressively longer, and seizure behaviours become more severe with repeated elicitation (Teskey, 2020). The afterdischarge threshold for each rat was determined by first stimulating the right BLA at 50 μA, and then increasing by 50 μA increments every 10 min (1 ms, biphasic square-wave pulse, 60 Hz for 1 s) until an afterdischarge lasting longer than 10 s was observed. Electrical stimulation was delivered twice daily, spaced 3 h apart for 10 consecutive days (20 total stimulations) at a stimulus intensity of 100 μA above each rats predetermined threshold to ensure an electrographic seizure was elicited. Sham groups undergo surgery and twice daily electrode hookups but are never stimulated. All behavioural seizures were scored using the Racine scale (Racine, 1972) and electrographic data taken from local field potential were recorded. The drugs administered throughout the study did not influence kindling progression: seizure duration (Fig. 1A), or seizure stage (Fig. 1B). Rats were considered fully kindled after three consecutive stage five seizures and only data from fully kindled rats were included in the analysis.

### 2.5 Blood collection and CORT ELISA

We first collected blood samples pre-kindling and then at 15, 30, 45-, 60-, 75- and 180-min post-seizure. Our results indicated that blood levels were maximal at 30 min post-seizure, similar to other acute stressors, and had returned to pre-kindling values by 180 min post-seizure.

Blood was collected 30 min following a seizure for CORT analysis in a separate cohort of rats at 6 predetermined time points throughout the experiment (before and after the 1st, 9th, and 19th elicited seizure). Collection times aligned with troughs in the biological rhythm for CORT to minimize variability between rats (De Boer and Van Der Gugten, 1987). Rats were placed into an acute restraint device (IBI Scientific) exposing the tail for venipuncture. Using a modified protocol adapted from (Lee and Goosens, 2015), the tail was immersed in warm water to dilate the lateral tail vein. Once identified, a small puncture was made laterally over the vein and approximately 1 mL of blood was collected into EDTA lined vacutainer tubes. Tubes were immediately centrifuged for 20 min at 14,000 RPM at 4 °C and plasma aliquoted and stored at −20 °C. Plasma corticosterone levels were quantified using a commercially available enzyme-linked immunosorbent assay (ELISA) kit (Arbor Assays), according to the manufacturer's guidelines. The detection limits for this kit based on the 100 μL standard used was between 5000 and 19.53 pg/mL. Every sample was diluted 1:1000 in the ELISA buffer so that values fell within the generated standard curve.

### 2.6 Elevated plus maze

One week following the last kindling session, rats were tested in a modified elevated plus maze (EPM) (Colangeli et al., 2020). Rats were habituated to the testing room by placing each home cage in illuminated, sound attenuating, and ventilated cabinets for 60 min before and after handling for 5 days prior to exposure on the EPM. Rats were handled on each of these days for 1 min. On testing day, rats were placed in the habituation cabinets for 60 min before EPM testing. The EPM system (Med Associates Inc.) is comprised of four arms of equal length (L:50 cm x W:10 cm), two of which had walls (L:50 cm x H:50 cm). Each arm was connected by a central platform (10 cm × 10 cm) that was not obstructed. The apparatus was housed in a sound-isolated room in red light conditions. Rats were placed on the central platform with the head oriented towards a closed arm. Exposure on the EPM lasted for 5-min, and all behaviours were recorded using a video camera and analyzed using the validated Noldus EthoVision XT 12.0.1138 (Ronquillo et al., 2023). Differences in locomotor activity was determined based on the total distance (cm) travelled. Anxiety-like behaviour was derived from the percentage of time spent in the open arms (time open %) expressed as [(time within open arms)/time within open and closed arms] x 100%; and the percentage of open arm entries (open entries %) expressed as [(number of open arm entries)/number of open and closed arm entries] x 100%.

### 2.7 Auditory fear conditioning

One week after the last kindling session, a separate cohort of rats were used to test for learned fear memory using an auditory fear conditioning paradigm. The experimental protocol and apparatus set-up was adapted, with minimal modifications (Morena et al., 2019; Colangeli et al., 2020). All fear conditioning chambers (30.5 x 24.1 × 21 cm^3^) were kept within sound attenuating and ventilated enclosures. The flooring of the chambers was composed of stainless-steel rods, which were connected to a shock generator, that allowed for timed delivery of shocks during testing. Context A chambers were comprised of metal walls on the back and the sides, a grid like pattern on the floor, a transparent Plexiglas front and ceiling, and white light. Context B chambers were constructed by covering the side metal walls and the grid flooring with white opaque plastic panels. Each context was thoroughly cleaned using ethanol (context A) or Virkon (context B) between rats. Rats were habituated to the testing room by placing each home cage in an illuminated, sound attenuating, and ventilated cabinet for 60 min before and after the handling on day one and two of the experiment, and for 90 min before and after testing, for the remaining days. On the first two days of the experiment, rats were individually habituated to the fear-conditioning chambers for a total of 10 min. Specifically, on day one context A and day 2, context B. On day three, auditory fear conditioning was performed in context A, which consisted of a 5-min acclimation period (Pre-CS) followed by three conditioning trials, for all rats. Each trial involved the presentation of a conditioned stimulus (CS; 80 dB, 4 Hz tone) for 30 s, immediately followed by a 1 s unconditioned stimulus (US; 0.90 mA footshock). The interval between CS-US pairing was 3 min. On day four, rats were evaluated on a 24-h fear memory retention schedule in context B, which involved the presentation of 18 CS (no US), with a 2-min interval between each successive CS. On day five, fear memory retention (48-h post-conditioning) was evaluated in context B, which involved the presentation of five CS (no US), also with an interval of 2 min. After all sessions, rats were returned to their home cage. All movement during the experiment was recorded using a video camera mounted on the inner door of the chamber. The video feed was analyzed for freezing behaviour (i.e., absence of movement except for respiration) using a validated video freeze software program (Med Associates Inc.) (Anagnostaras et al., 2010; Zelikowsky et al., 2012). The videos were secondarily manually scored to observe the presence of relaxed behaviours (i.e., tail, forelimbs, and hindlimbs resting on the floor, or asleep) that may be misinterpreted for freezing by the video software. Encountering relaxed behaviours is a rare event (<2%) and the rats (3) that displayed this behaviour were removed from all experimental groups.

### 2.8 Endocannabinoid analysis

One week after the last seizure or sham stimulation, rats were euthanized by rapid decapitation. Both the left and right amygdala were dissected and stored at −80 °C until tissue processing. The extraction and quantification process was conducted as previously described (Hill et al., 2010a; Qi et al., 2015). In brief, amygdala tissue was placed into borosilicate glass culture tubes containing 2 mL of acetonitrile (ACN), 5 pmol of [2H8] AEA, and 5 nmol of [2H8] 2-AG. The solution was sonicated for 30 min in an ice water bath and then left to incubate overnight in −20 °C to allow protein precipitation before centrifugation. The lipid containing solution was transferred to a new glass tube and evaporated using nitrogen gas. Following a second reconstitution and drying step the sample was diluted in 20 μL of ACN and stored at −80 °C. Analysis of AEA and 2-AG was performed by liquid chromatography tandem mass spectrometry in the University of Calgary Mass Spectroscopy Facility.

### 2.9 Electrophysiology

Whole cell patch clamp recording from pyramidal neurons of the BLA was performed as previously described (Colangeli et al., 2020, 2023). Briefly, one week after the last sham stimulation or kindled seizure, coronal brain slices of the BLA (300 μm) were prepared with a vibratome (model VT1200S, Leica) in ice-cold slicing solution containing the following (in mM): 87 NaCl, 2.5 KCl, 25 NaHCO3, 0.5 CaCl2, 7 MgCl2, 1.25 NaH2PO4, 25 d-glucose, and 75 sucrose. Slices were then incubated in a holding chamber with oxygenated aCSF containing the following (in mM): 126 NaCl, 2.5 KCl, 2.5 CaCl_2_, 1.5 MgCl_2_, 1.25 NaH_2_PO_4_, 26 NaHCO_3_, and 10 d-glucose, at pH 7.4 for 30 min at 32 °C. After at least 1 h of incubation, slices were placed in the recording chamber and continuously superfused with regular aCSF at a flow rate of 1.5 ml/min at room temperature. Intrinsic membrane properties and spontaneous excitatory postsynaptic currents (sEPSC) were recorded with borosilicate glass electrodes (4–6 MΩ) filled with the standard intracellular solution with the following composition (in mM): 108 K-gluconate, 8 Na-gluconate, 2 MgCl2, 8 KCl, 4 K2-ATP, 0.3 Na-GTP, 1 EGTA, and 10 HEPES, pH 7.4 adjusted with KOH. EPSCs were recorded at the Cl- (−60 mV) reversal potential. GABA_A_ receptor-mediated spontaneous inhibitory post-synaptic currents (sIPSCs) were recorded by using glass electrodes (3–5 MΩ) filled with a solution containing the following (in mM): 90 CsCH_3_SO_3_, 50 CsCl, 1 EGTA, 10 HEPES, 4.6 MgCl_2_, 0.1 CaCl_2_, 5 QX314, 0.3 Na-GTP, and 4 Mg-ATP, at pH 7.3 adjusted with CsOH. sIPSCs were isolated by adding to the bath 40 μM d-AP-5 and 20 μM DNQX to block glutamatergic transmission. The amplitudes and frequencies of sIPSCs/sEPECs were detected (and confirmed by visual inspection) and analyzed from 300 continuous seconds of recording by Mini Analysis 6.0.7 software (Synaptosoft) using thresholds of five times rms noise levels. Neurons were held at −60 mV to record sEPSCs and −70 mV to record sIPSCs. Cell capacitance and access resistance (initial value, <30 MΩ) were monitored during experiments, and recordings were accepted for analysis if either variable did not change by >20%. Signals were collected via an Axopatch 200B amplifier (Molecular Devices). The pCLAMP 9 software (Molecular Devices) was used for data acquisition.

### 2.10 Statistical analysis

All statistical analyses were performed using Prism 10.0.2 (GraphPad, La Jolla, Ca). Power analyses were performed for each experiment after pilot data was collected to determine appropriate sample size. Two-way and repeated measures ANOVAs followed by Tukey's post hoc tests were applied to determine multiple comparison. Comparisons between two unpaired groups were analyzed using a Student's t-test. Data were tested for homogeneity of variance between groups using Levene's test. Normality was assessed for each comparison using the Shapiro-Wilk test. Thresholds for both tests were set to p = 0.05 and neither parameter was violated. Significance threshold was accepted at p < 0.05 and statistical design are outlined in each figure caption.

## 3 Results

### 3.1 Kindled seizures of the BLA produce a robust increase in CORT which is prevented by metyrapone

**Fig. 2:**
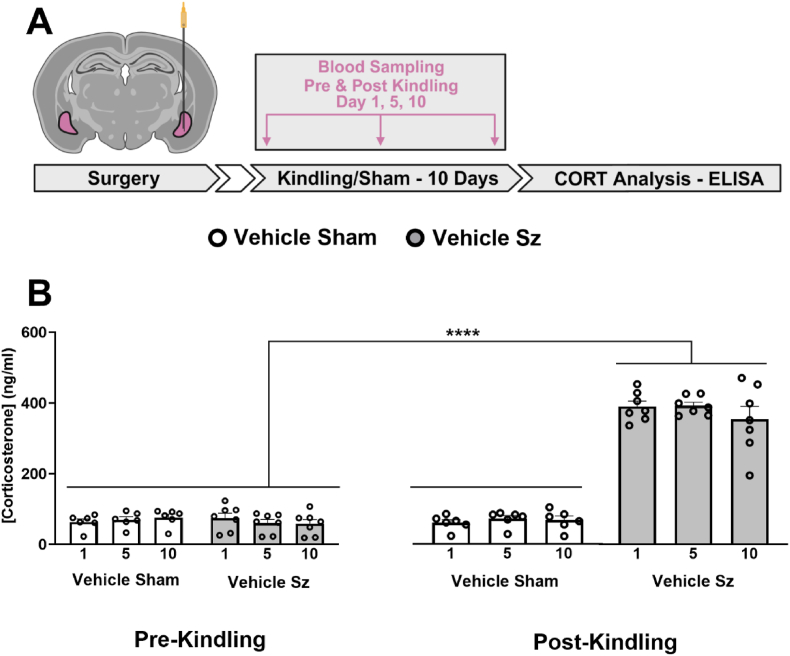
**Kindled seizures produce a large increase in CORT**. Kindled seizures generate a large CORT response that does not habituate over repeated sessions. These changes could be prevented using the 11-betahydroxylase inhibitor, metyrapone (50 mg/kg; data not shown). **A)** Experimental schematic illustrating the timeline of CORT extraction/analysis experiments. White filled arrows represent a 7-day break from experimentation. **B)** Kindled BLA seizures trigger a significant elevation in the stress hormone, CORT (two-way RM ANOVA, seizure effect: F_(1,22)_ = 39.31, p < 0.0001; time effect: F_(1,22)_ = 48.84, p < 0.0001; Interaction: F_(1,22)_ = 48.56, p < 0.0001). Rats do not habituate to the seizure-induced increases in CORT as there are no differences measured over kindling days (two-way RM ANOVA, day effect: F_(2,33)_ = 0.61, p = 0.55; Interaction: F_(2,33)_ = 0.70, p = 0.51). The increase in CORT following each kindled seizure is completely abolished using metyrapone (data not shown). Overall ANOVA statistics are reported by comparing day 10 of each experimental group. Significance in figures is represented using a Tukey's multiple comparisons follow up test. Data are expressed as means ± SEM. ∗∗∗∗p < 0.0001; n = 6–7 per group. Graphics created with Biorender.com.

Kindling, a robust animal model of temporal lobe epilepsy used in the present study recapitulates many core features of the disease including a rapid sensitization to the stimuli, and the dysregulation of neural circuitry leading to emotional and cognitive impairments (Corcoran and Teskey, 2009; Kanner, 2016; Teskey, 2020). To determine whether seizures in the BLA elicit systemic CORT response over the course of kindling, blood was collected before and after a sham or kindled seizure, the first (day 1), ninth (day 5) and nineteenth (day 10) of twenty kindled seizures (Fig. 2A). A single kindled seizure triggered a large and significant increase in CORT that was equivalent over the first, fifth and tenth day indicating that the seizure-induced increase in CORT levels did not habituate over subsequent sessions (Fig. 2B). When metyrapone, a potent CORT synthesis inhibitor was administered prior to each seizure, the seizure-induced elevated corticosterone response following each seizure was abolished (data not shown).

### 3.2 Pharmacologically ablating seizure-induced CORT signaling with metyrapone prevents emotional dysfunction

**Fig. 3:**
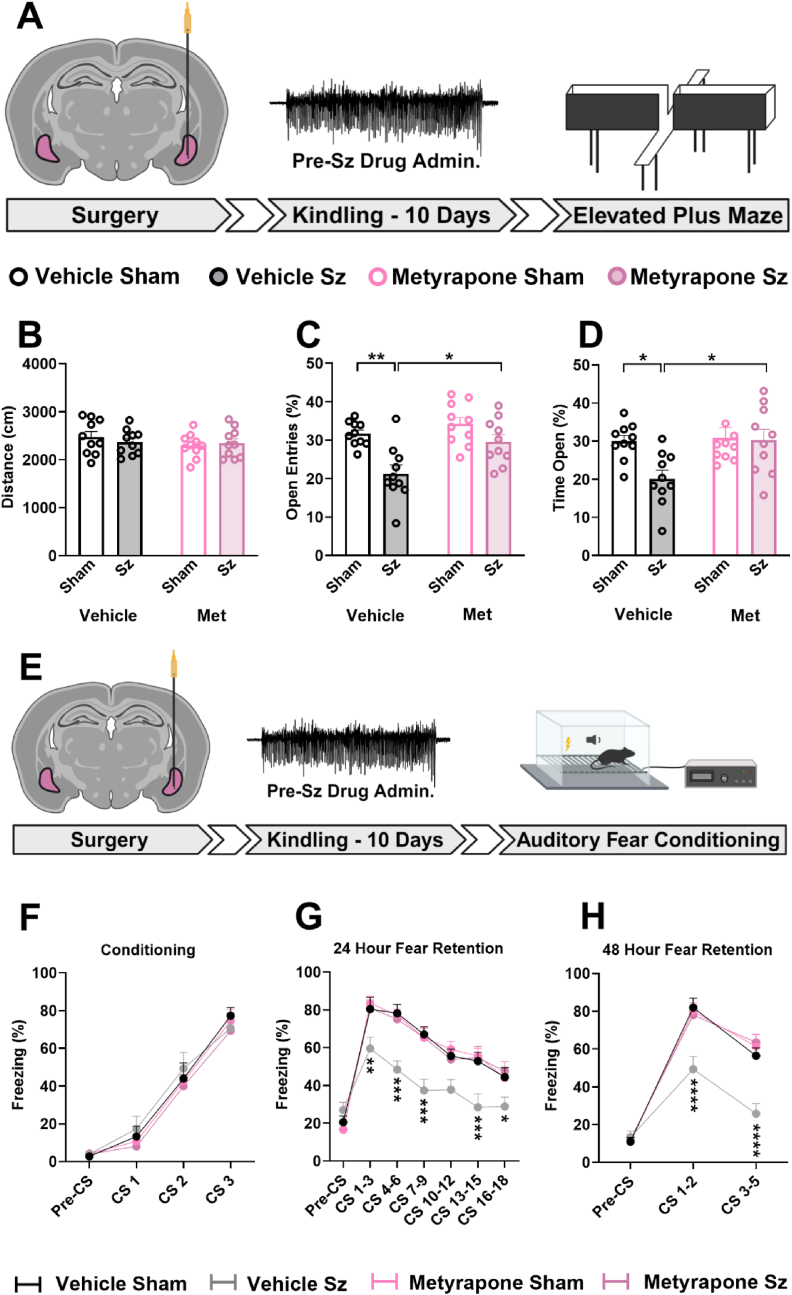
**Metyrapone prevents seizure-induced emotional dysfunction**. Kindled rats display both an anxious-like phenotype and have impaired fear memory retention measured 24- and 48-h post conditioning; these behaviours are reverted when metyrapone is administered prior to each kindled seizure. **A)** Experimental schematic illustrating the timeline of elevated plus maze experiments. White filled arrows represent a 7-day break from experimentation. **(B-D)** Kindled BLA seizures promote anxious-like behaviours on the EPM which are prevented with pre-seizure metyrapone administration **B)** Effects of metyrapone administration and repeated kindled seizures on total distance travelled, (two-way ANOVA, seizure effect; F_(1,36)_ = 0.10, p = 0.75; drug effect: F_(1,36)_ = 1.21, p = 0.28; interaction: F_(1,36)_ = 0.67, p = 0.42), **C)** percentage of time spent in the open arms (two-way ANOVA, seizure effect: F_(1,36)_ = 4.71, p = 0.037; drug effect: F_(1,36)_ = 5.28, p = 0.028; interaction: F_(1,36)_ = 3.79, p = 0.060), and **D)** percentage of open arm entries (two-way ANOVA, seizure effect: F_(1,36)_ = 17.85, p = 0.0002; drug effect: F_(1,36)_ = 9.31, p = 0.0043; interaction: F_(1,36)_ = 2.53, p = 0.12). **E)** Experimental schematic illustrating the timeline of auditory fear conditioning experiments. White filled arrows represent a 7-day break from experimentation. **(F-H)** Kindled seizures impair fear memory behaviours at 24- and 48-h post-conditioning; these effects were fully prevented using metyrapone**. F)** Effects of metyrapone administration and repeated kindled seizures on freezing rates expressed as a percent during cued fear conditioning (two-way RM ANOVA, seizure effect: F_(3,224)_ = 0.62, p = 0.62; CS effect: F_(3,224)_ = 133.3, p < 0.0001; interaction: F_(3,224)_ = 0.2943, p = 0.98), **G)** 24-h retention (two-way RM ANOVA, seizure effect: F_(3,392)_ = 27.37, p < 0.0001; CS effect: F_(6,392)_ = 62.24, p < 0.0001; interaction: F_(18,392)_ = 2.038, p = 0.0075), and **H)** 48-h retention protocols (two-way RM ANOVA, seizure effect: F_(3,168)_ = 16.21, p < 0.0001; CS effect: F_(2,168)_ = 182.00, p < 0.0001; interaction: F_(6,168)_ = 5.11, p < 0.0001). Significance in figures is represented using a Tukey's multiple comparisons follow up test. Post-hoc analyses on fear retention graphs compare vehicle seizure to metyrapone seizure. Data are expressed as means ± SEM. ∗p < 0.05, ∗∗p < 0.01, ∗∗∗p < 0.001, ∗∗∗∗p < 0.0001; n = 10–15 per group. Graphics created with Biorender.com.

We then determined whether amygdala kindling in our model persistently altered emotional behaviour. We measured anxiety-like behaviours on the EPM in sham or kindled rats one week following the last elicited seizure (Fig. 3A). The EPM is a validated model used to assess anxiety-related behaviour in rodents. No differences were observed in locomotor activity between groups over the duration of the task (Fig. 3B). As previously reported (Colangeli et al., 2020), here we also observed that repeated amygdala kindling leads to a significant increase in anxiety-like behaviours as represented by both the reduced percentage of the number of open entries (Fig. 3C) and the reduced percentage of the total time spent (Fig. 3D) within the open arms displayed in rats subjected to kindling with respect to the sham group. Kindled rats repeatedly administered with metyrapone before each seizure showed comparable percentages of entries (Fig. 3C) and open arm exposure (Fig. 3D) to sham rats given either vehicle or drug when tested one week after the last evoked seizure. These data provide evidence that repeated prevention of seizure-induced CORT surge completely prevented the seizure-induced alteration of anxiety-like behaviour in the EPM.

In a separate cohort of rats, the long-term effect of repeated amygdala seizures on fear memory dynamics were assessed (Fig. 3E). To understand the mechanisms of associative memory as they pertain to emotional dysfunction, we employed a classical contextual fear conditioning paradigm. Rats subjected to sham or kindled seizure were tested in the fear conditioning paradigm one week after the last evoked seizure in a seizure and drug free condition. During the acquisition of the fear response, we observed no differences in freezing behaviours between groups during CS-US pairings, showing that all groups make the association appropriately (Fig. 3F). Rats that were kindled and treated with vehicle showed impaired fear memory retention at both 24-h (Fig. 3G) and 48-h (Fig. 3H) as represented by a significant reduction in freezing behaviour during the conditioned stimulus (CS-tone) presentations compared to other groups. Rats that were previously administered with metyrapone prior to each seizure displayed preserved fear memory retention, as freezing behaviour during CS presentation were no longer statistically different with respect to both sham groups at 24- and 48-h post-acquisition (Fig. 3G–H). Collectively, these data provide evidence that repeated CORT response drives emotional dysfunction associated with seizure activity.

### 3.3 Blocking corticosterone synthesis prevents the seizure-induced alteration in AEA content and glutamatergic and GABAergic transmission

**Fig. 4:**
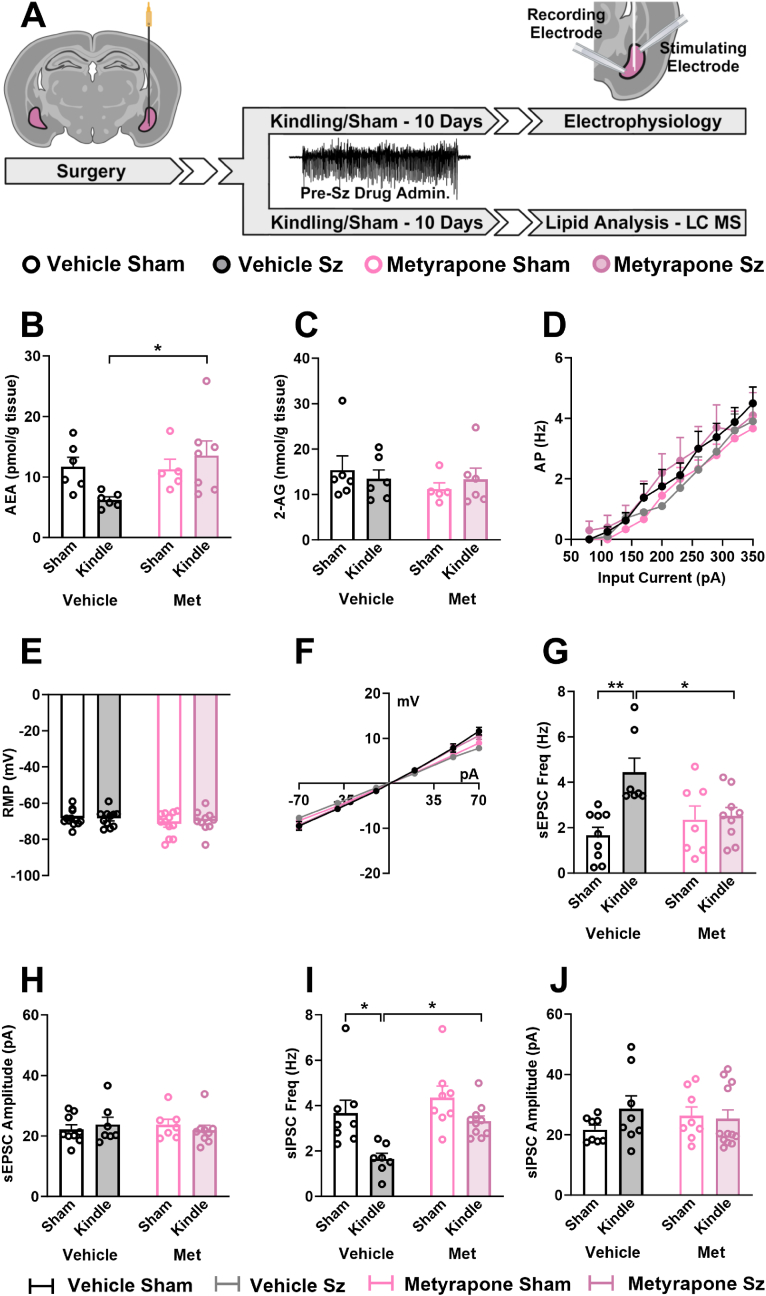
**Metyrapone rescues AEA levels and restores the seizure-induced imbalance of glutamate/GABA activity onto BLA pyramidal neurons**. Repeated kindled seizures cause a downregulation in AEA content that is paralleled by an imbalance in both excitatory and inhibitory presynaptic activity within the BLA. **A)** Experimental schematic illustrating the timeline of eCB analysis and electrophysiological experiments. White filled arrows represent a 7-day break from experimentation. **B/C)** Kindled seizures cause a persistent drop in AEA but not 2-AG that can be fully restored using metyrapone. **B)** Anandamide (two-way ANOVA, seizure effect: F_(1,20)_ = 3.60, p = 0.26; drug effect: F_(1,20)_ = 0. 79, p = 0.38; interaction: F_(1,20)_ = 4.51, p = 0.046), and **C)** 2-Arachidonyl Glycerol (two-way ANOVA, seizure effect: F_(1,19)_ = 0.0021, p = 0.96; drug effect: F_(1,19)_ = 0.77, p = 0.39; interaction: F_(1,19)_ = 0.69, p = 0.42). **D)** Relationship of evoked action potential to increasing current steps was similar across all experimental groups (two-way RM ANOVA, seizure effect: F_(3,330)_ = 3.71, p = 0.012; current effect: F_(9,330)_ = 41.17, p < 0.0001; interaction: F_(27,330)_ = 0.21, p > 0.99); n = 9–12 cells, 8–10 rats. **E)** The resting membrane potential between all experimental groups was similar: Vehicle No Sz (−68.41 ± 1.29), Vehicle Sz (−68.44 ± 1.39), Metyrapone No Sz (−71.47 ± 1.86), Metyrapone Sz (−69.92 ± 1.90); n = 10–12 cells, 9–10 rats. **F)** I-V curves showing similar responses between all experimental groups (two-way RM ANOVA, seizure effect: F_(3,266)_ = 0.059, p = 0.98; current effect: F_(6,266)_ = 622.5, p < 0.0001; interaction: F_(18,266)_ = 3.011, p < 0.0001); n = 9–12 cells, 8–10 rats. **(G/H)** Repeated kindled seizures significantly increase excitatory presynaptic activity without changing the amplitude of the response. Metyrapone administration restored the seizure-induced increase in sEPSC frequency **(G**, n = 7–9 cells, 6–7 rats; two-way ANOVA, seizure effect: F_(1,28)_ = 9.732, p = 0.0048; drug effect: F_(1,28)_ = 1.64, p = 0.21; interaction: F_(1,28)_ = 7.51, p = 0.011) without any significant effect on sEPSC amplitude **(H**, n = 7–9 cells, 7 rats; two-way ANOVA, seizure effect: F_(1,28)_ = 0.0060, p = 0.94; drug effect: F_(1,28)_ = 0.0062, p = 0.94; interaction: F_(1,28)_ = 0.85, p = 0.036). **(I/J)** Repeated kindled seizures significantly reduces inhibitory presynaptic activity without changing the amplitude of the response. Metyrapone administration reversed the seizure-indued decrease in sIPSC frequency **(I**, n = 7–10 cells, 7–9 rats; two-way ANOVA, seizure effect: F_(1,29)_ = 13.39, p = 0.0010; drug effect: F_(1,29)_ = 7.92, p = 0.0087; interaction: F_(1,29)_ = 1.39, p = 0.25) without any significant effect on sIPSC amplitude **(J**, n = 8–12 cells, 7–11 rats; two-way ANOVA, seizure effect: F_(1,32)_ = 0.86, p = 0.36; drug effect: F_(1,32)_ = 0.044, p = 0.83; interaction: F_(1,32)_ = 1.55, p = 0.22). Significance in figures is represented using a Tukey's multiple comparisons follow up test. Data are expressed as means ± SEM. ∗p < 0.05, ∗∗p < 0.01. Graphics created with Biorender.com.

Repeated kindled seizures have been recently reported to downregulate AEA levels within the rat amygdala and by pharmacologically enhancing AEA signaling, proper BLA synaptic function and emotional behaviour could be restored in epileptic rats (Colangeli et al., 2020). Thus, to test a possible downstream mechanism by which seizure-induced activation of the HPA axis drives alterations in emotionality in kindled rats we first assessed eCB levels in the BLA in rats subjected to either sham or kindled seizure and treated with vehicle or metyrapone (Fig. 4A). One week following the last kindled seizure, AEA levels were measured and found to be significantly lower in rats that were kindled compared to sham rats. Repeated pre-seizure metyrapone treatment prevented the seizure-induced reduction in AEA levels when assessed one week after the last of the twenty evoked seizures (Fig. 4B). There were no changes in the other contributing eCB, 2-arachidonoylglycerol (2-AG), resulting from seizures or metyrapone treatment (Fig. 4C).

We then tested whether the reduced AEA content in the amygdala was associated with altered cellular excitability and synaptic function of BLA pyramidal neurons.

Previous kindling studies reported no alteration of the intrinsic membrane properties of BLA neurons following repeated seizure activity (Shoji et al., 1998; Colangeli et al., 2020). Here we also found no differences in the current-voltage (I-V) relationship (Fig. 4D), the resting membrane potentials (Fig. 4E), or the number of action potentials generated in response to increasing current steps (Fig. 4F) between any of the experimental groups. These data confirm the absence of changes on basal neuronal excitability in BLA pyramidal neurons by seizure induction and repeated CORT surge.

To evaluate changes in synaptic transmission within the BLA following seizures, we assessed basal excitatory and inhibitory activity onto BLA pyramidal neurons one week after the last seizure. Consistent with previous findings (Colangeli et al., 2020), here we observed a significant increase in the frequency of sEPSC of kindled rats that were treated with vehicle, while repeated metyrapone treatment significantly prevented sEPSCs frequency increase, such that there were no differences when compared to both sham groups (Fig. 4G). There were no differences in the amplitude (Fig. 4H) of sEPSC recorded from BLA pyramidal cells in any of the experimental groups indicating that changes in synaptic strength performed by stress hormones released during seizures might be restricted to the pre-synaptic site of excitatory terminals onto BLA pyramidal neurons. In parallel, we observed a significant reduction in the frequency (Fig. 4I), but not amplitude (Fig. 4J) of sIPSCs in rats that were kindled and given vehicle. Pre-seizure treatment with metyrapone resulted in typical GABAergic transmission to frequencies expressed in seizure-free rats. Altogether, metyrapone prevented the seizure-induced reduction in AEA levels and alterations in both glutamatergic and GABAergic transmission within the BLA.

### 3.4 Inhibiting both glucocorticoid and mineralocorticoid receptors raises baseline CORT levels without affecting the seizure induced CORT surge

**Fig. 5:**
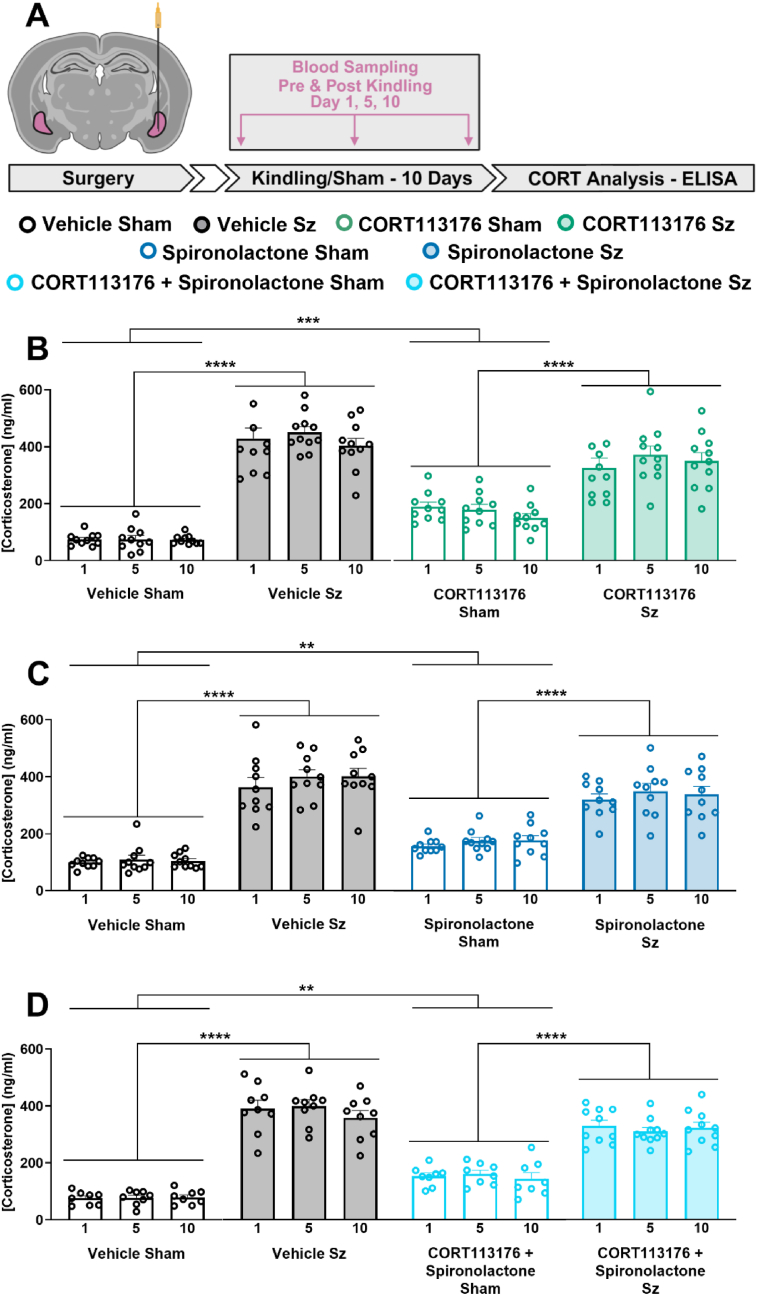
**The seizure-induced CORT response remains after glucocorticoid and mineralocorticoid receptor antagonism**. Selective pharmacological blockade of GR and MR does not prevent the seizure-induced increase in corticosterone. **A)** Experimental schematic illustrating the timeline of corticosterone extraction/analysis experiments. White filled arrows represent a 7-day break from experimentation. **B)** Pre-seizure administration of CORT113176 significantly raised baseline CORT levels but did not reduce the rapid CORT increase in response to seizures (two-way RM ANOVA, seizure effect: F_(1,38)_ = 145.60, p < 0.0001; drug effect: F_(1,38)_ = 0.24, p = 0.63; interaction: F_(1,38)_ = 8.83, p = 0.0051). **C)** Pre-seizure administration of spironolactone significantly raised baseline CORT levels but did not reduce the rapid CORT increase in response to seizures (two-way RM ANOVA, seizure effect; F_(1,36)_ = 109.10, p < 0.0001; drug effect: F_(1,36)_ = 0.053, p = 0.82; interaction: F_(1,36)_ = 9.43, p = 0.0040. **D)** Co-administration of CORT113176 and spironolactone also raised CORT levels at baseline without changing the seizure-induced CORT response (two-way RM ANOVA, seizure effect: F_(1,31)_ = 127.00, p < 0.0001; drug effect: F_(1,31)_ = 0.53, p = 0.47; interaction: F_(1,31)_ = 6.08, p = 0.02). Overall ANOVA statistics are reported by comparing day 10 of each experimental group. Significance in figures is represented using a Tukey's multiple comparisons follow up test. Data are expressed as means ± SEM. ∗∗p < 0.01, ∗∗∗p < 0.001, ∗∗∗∗p < 0.0001; n = 8–11 per group. Graphics created with Biorender.com.

Here we determined if repeated inhibition of GR and MR altered basal CORT production; an important consideration if these drugs are to be used to prevent the consequences of the seizures-induced CORT surge. We administered the selective GR antagonist CORT113176 alone, the MR antagonist spironolactone alone, or both antagonists together 30 min prior to sham or seizure elicitation over the kindling protocol (Fig. 5A). Pre-seizure administration of either or both antagonists raised baseline CORT at all timepoints suggesting that antagonism at both receptors disrupt the typical CORT negative feedback response. Importantly, CORT113176 administered alone (Fig. 5B) or spironolactone administered alone (Fig. 5C) did not change the elevated CORT response to seizures relative to kindled rats treated with vehicle. A comparable CORT response was observed between each experimental group when CORT113176 was given in combination with spironolactone (Fig. 5D).

### 3.5 Glucocorticoid and mineralocorticoid receptor antagonists prevent seizure-induced emotional behaviour

Given that the prevention of CORT synthesis before each seizure using metyrapone prevented the persistent higher anxiety-like behaviour and altered fear memory retention in kindled rats, we asked a similar question using a potentially translational approach by inhibiting GR and MR to prevent emotional dysregulation.

**Fig. 6:**
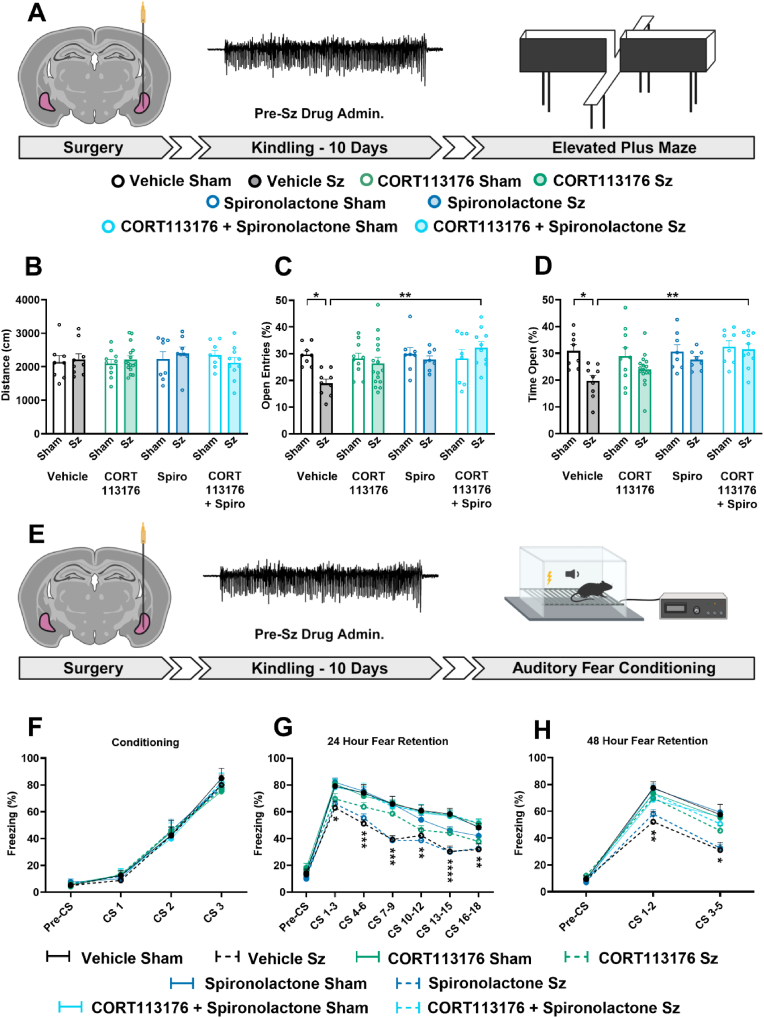
**CORT113176 when co-administered with spironolactone prevents seizure-induced emotional dysfunction**. CORT11376 and Spironolactone (but not CORT113176 alone or spironolactone alone) given together before each electrically kindled seizure reverted the chronic dysfunctions observed on the elevated plus maze and fear processing paradigms. **A)** Experimental schematic illustrating the timeline of elevated plus maze experiments. White filled arrows represent a 7-day break from experimentation. **(B-D)** Co-administration of CORT11376 and Spironolactone prevented the increase in seizure-induced anxious-like behaviours on the EPM. **B)** Effects of GR and MR antagonism and repeated kindled seizures on total distance travelled, (two-way ANOVA, seizure effect: F_(1,69)_ = 0.11, p = 0.74; drug effect: F_(3,69)_ = 0.41, p = 0.74; interaction: F_(3,69)_ = 0.65, p = 0.59), **C)** percentage of time spent in the open arms (two-way ANOVA, seizure effect; F_(1,69)_ = 10.19, p = 0.0021; drug effect: F_(3,69)_ = 3.51, p = 0.020; interaction: F_(3,69)_ = 1.85, p = 0.15), and **D)** percentage of open arm entries (two-way ANOVA, seizure effect: F_(1,69)_ = 2.66, p = 0.11; drug effect: F_(3,69)_ = 2.30, p = 0.085; interaction: F_(3,69)_ = 3.34, p = 0.024). **E)** Experimental schematic illustrating the timeline of auditory fear conditioning experiments. White filled arrows represent a 7-day break from experimentation. **(F-H)** Co-administration of CORT11376 and Spironolactone prevented the seizure-induced impairment of fear behaviours measured 24- and 48-h post-conditioning**. F)** Effects of GR and MR antagonism and repeated kindled seizures on freezing rates expressed as a percent during cued fear conditioning (two-way RM ANOVA, seizure effect: F_(7,328)_ = 0.078, p = 0.99; CS effect: F_(3,328)_ = 356.20, p < 0.0001; interaction: F_(21,328)_ = 0.22, p > 0.99). **G)** 24-h retention (two-way RM ANOVA, seizure effect: F_(7,574)_ = 28.27, p < 0.0001; CS effect: F_(6,574)_ = 187.80, p < 0.0001; interaction: F_(42,574)_ = 1.21, p = 0.18), and **H)** 48-h retention protocols (two-way RM ANOVA, seizure effect: F_(7,246)_ = 7.12, p < 0.0001; CS effect: F_(2,246)_ = 422.00, p < 0.0001; interaction: F_(14,246)_ = 2.11, p = 0.012). Significance in figures is represented using a Tukey's multiple comparisons follow up test. Post-hoc analyses on fear retention graphs compare vehicle seizure to CORT113176+Spironolactone seizure. Data are expressed as means ± SEM. ∗p < 0.05, ∗∗p < 0.01, ∗∗∗p < 0.001, ∗∗∗∗p < 0.0001; n = 8–18 per group. Graphics created with Biorender.com.

We first determined whether a systemic inhibition of GR and MR prior to each evoked sham or kindled seizure would prevent altered emotionality evaluated on the EPM one week after the last seizure (Fig. 6A). We observed no differences in total distance travelled across each of the treatment groups indicating that CORT receptor activity did not alter locomotor activity (Fig. 6B). When CORT113176 and spironolactone were administered alone prior to each seizure, we did observe in kindled rats an increased percentage of open arm entries (Fig. 6C) and percentage of time spent in the open arms (Fig. 6D) with respect to vehicle treated kindled rats albeit not significant. When rats were treated simultaneously with both antagonists, kindled rats displayed percentage of open arm entries (Fig. 6C) and percentage of time spent in the open arms (Fig. 6D) not statistically different from sham rats, thus demonstrating that both MR and GR are required for CORT induced changes in anxiety-like behaviour.

Finally, we asked whether antagonism of either receptor was able to prevent the seizure-induced impairment of fear memory dynamics (Fig. 6E). Similar to metyrapone, there was no change in the freezing response during CS- unconditioned stimulus (US-shock) pairings among groups, (Fig. 6F). Kindled rats that were administered vehicle displayed reduced freezing behaviour at 24- and 48-h post-conditioning. When kindled rats were treated with CORT113176 or spironolactone administered alone they showed no statistically different freezing response at either timepoint, with respect to kindled rats treated with vehicle. On the other hand, antagonism of both the GR and MR prevented fear memory alteration in rats that were kindled as freezing behaviour during presentations of the CS were not statistically different to those of the sham groups at 24-h (Fig. 6G) and 48-h (Fig. 6H). These data provide evidence that CORT mediates changes in behavioural outcomes via action at both steroid receptor targets.

## 4 Discussion

In the present study and for the first time, we provide evidence that electrically kindled seizures produce a robust CORT response that is paralleled with persistent downregulation of amygdala AEA but not 2-AG content, BLA neuronal hyperexcitability, higher anxiety-like behaviour and impaired fear memory processing in rats. We show that the inhibition of CORT synthesis prevented amygdala AEA decline and electrophysiological and behavioural dysfunction in rats that were fully kindled. We then provide evidence that both GRs and MRs are required for the development of seizure-induced alterations in emotional behaviour. Thus, we provide a target to treat the maladaptive physiological and behavioural response observed in people with epilepsy.

Stress and epilepsy have been shown to have a bidirectional relationship as stress related hormones are capable of lowering seizure threshold (Kraus et al., 2023) and, seizure expression is capable of increasing the activity of the HPA axis (Wulsin et al., 2016). Clinical and preclinical studies have demonstrated that elevated glucocorticoid levels or over activation of the HPA axis is often associated with the pathophysiology of many psychiatric disorders (Tsigos et al., 2000; Lupien et al., 2009; Hill and Spencer-Segal, 2021). Our model provides temporally correlated evidence that electrically kindled seizures produce a transitory generalized CORT response that is responsible for the negative consequences of seizure activity.

Habituation to stressful stimuli is an adaptive response that is highly conserved across repeated homotypic stressors as a mechanism to reduce the negative physiological response (Grissom and Bhatnagar, 2009; Morena et al., 2016). The innate ability to habituate to non-threatening stimuli is, in part regulated by CORT negative feedback mechanisms since inhibiting GRs and/or MRs has been shown to prevent habituation to homotypic stress (Cole et al., 2000; Jaferi and Bhatnagar, 2006). Here, we show that the response to seizure-induced CORT production is maximal in keeping with other reports following an acute stressor (Atrooz et al., 2021) and does not habituate with repeated kindled seizures and is likely a result of HPA activation performed by principal output nuclei of the amygdala where seizures were induced. Indeed, the evoked CORT response on day one where the seizure is confined (focal) is comparable to day ten when a widespread (fully generalized) seizure is observed. Pre-seizure treatment with metyrapone completely abolished the CORT surge without disrupting baseline CORT; an effect that has previously been reported (Verhelst et al., 1991; Kvarta et al., 2015). Our experiments with metyrapone provide a proof-of-principle that seizure-induced amygdala hyperactivity leads to an uncontrolled HPA activation to drive seizure-induced behavioural dysfunction.

To determine the molecular targets required for the behavioural effects of the seizure-induced CORT surge, we inhibited the GRs and MRs. Here, we demonstrated that both GR and MR activity facilitate the behavioural deficits associated with seizure-induced stress. Indeed, antagonizing GRs alone or MRs alone was not sufficient to prevent behavioural changes associated with seizures, rather, the simultaneous inhibition of GRs and MRs was required. Both GRs and MRs independently, when antagonized, have been reported to dampen the behavioural response to a stressor (Sapolsky et al., 2000; Hammerslag et al., 2021; Albernaz-Mariano and Munhoz, 2022; de Azevedo Camin et al., 2022). While some effects of stress have been attributed to MR and GR activity in isolation, CORT acts at both MRs and GRs (Hartmann et al., 2019; Kloet et al., 2019), prompting investigation into a multi receptor approach to better understanding seizure-induced stress signaling. We demonstrate the therapeutic value of corticosteroid antagonism in seizure/stress-related psychopathology. However, a systemic approach to inhibiting these integral receptors can lead to unfavorable effects resulting from a disrupted CORT negative feedback and subsequent sustained increased CORT levels. Indeed, our experiments in conjunction with others who have utilized specific GR and MR antagonists, show a significant increase in basal CORT likely because of impaired feedback inhibition at regulatory checkpoints along the HPA axis (Zalachoras et al., 2013; Gehrand et al., 2021, 2022). While these selective drugs show promise in preventing the emotional dysfunction that follow repeated kindled seizures the effect of raised CORT levels and potential consequences resulting from chronic consumption such as the effects on other organs needs to be considered.

Stress and CORT strongly modulate eCB neurotransmission, efficacy, and availability (Hill et al., 2005, 2010c; Morena et al., 2016; Balsevich et al., 2017). Repeated stress or CORT exposure have been shown to downregulate AEA availability across multiple brain regions including the amygdala (Rademacher et al., 2008; Hill et al., 2010b) leading to maladaptive behavioural responses to stressors (Morena et al., 2014, 2015, 2016). In line with this evidence augmentation of AEA signaling is a promising target for the treatment of stress-related psychiatric disorders (Patel et al., 2017). Similarly, this parallels observations that show repeated kindled seizures also trigger dysregulation of AEA signaling (Colangeli et al., 2020, 2021, 2023; Farrell et al., 2021). In particular AEA signaling deficiency seems to play a crucial role in dysfunctional emotional behaviour in the epileptic condition in both nonhuman animal and human studies (Colangeli et al., 2020; Rocha et al., 2020; Epps, 2022; Martínez-Aguirre et al., 2022). Here, we show a significant drop in AEA as a chronic consequence of repeated seizure-induced stress-like response. When CORT synthesis was blocked with metyrapone, AEA levels remained unchanged, and this was associated with proper emotional behaviour. Here we propose that seizure-induced CORT surge is a potential modulator of long-term downregulation of AEA content in BLA to drive alteration of emotional behaviour in epileptic rats. Indeed, either prevention of repeated seizure-induced CORT response (found here) or pharmacologically restoration of AEA availability following seizure activity (Colangeli et al., 2020) are equally effective in rescuing the behavioural deficits observed in kindled animals.

At the cellular level, both stress and the eCB systems function bidirectionally through the modulation of synaptic activity in brain regions involved in emotional behaviours (Senst and Bains, 2014; Morena et al., 2016). Stress and stress-related hormones have been shown to persistently increase excitatory transmission in the amygdala (Karst et al., 2010; Rosenkranz et al., 2010; Popoli et al., 2011; Wilson et al., 2015), an effect that triggers emotional dysregulation associated with a stress exposure (Tye et al., 2011; Wilson et al., 2015; Marcus et al., 2020). The eCB system tightly controls synaptic activity by providing regulatory checkpoints at both excitatory and inhibitory cell types (Castillo et al., 2012; Kano, 2014). Disrupted AEA tone within the principal neurons of the BLA has been shown to dramatically contribute to BLA hyperexcitability, anxiety and emotional memory alteration (Moreira et al., 2008; Romigi et al., 2010; Bluett et al., 2014; Gray et al., 2015; Colangeli et al., 2020; Yasmin et al., 2020). We have previously reported that in naïve rats, excitatory terminals onto BLA pyramidal neurons are under an inhibitory eCB tone (Colangeli et al., 2020). Moreover, reduced amygdala AEA levels in kindled rats parallel the absence of the eCB inhibitory tone over glutamatergic terminals and increased glutamate transmission in the BLA (Colangeli et al., 2020). Since, both the reduced amygdala AEA levels and the increased excitatory drive onto BLA pyramidal cell are prevented by metyrapone administration, we postulate that the seizure-induced CORT surge mimics a stress-like response that impairs AEA inhibitory control over glutamatergic terminals leading to BLA hyperexcitability. Corroborating our hypothesis, pharmacological inhibition of the fatty acid amid hydrolase (FAAH) enzyme has previously been shown to dampen the increased excitatory transmission in the BLA induced by both stress exposure (Yasmin et al., 2020) and kindling (Colangeli et al., 2017, 2020).

The other main eCB, 2-AG, plays a crucial role in the amygdala physiology and emotional behaviour. 2-AG signaling is highly involved in both seizure termination (Farrell et al., 2021) and termination and adaptation of the HPA axis during stress (Morena et al., 2016). In addition, it has been reported an intimate pharmacological redundancy between AEA and 2-AG signaling in the BLA, such that augmentation of 2-AG can compensate for stress-induced deficiencies in AEA (Bedse et al., 2017). Thus, despite the fact that tonic inhibitory control of presynaptic CB1 activity onto BLA glutamatergic transmission appears to be mediated by AEA rather than 2-AG signaling (Yasmin et al., 2020), and 2-AG content in the amygdala is not altered following seizure expression (our current data and Colangeli et al., 2020), the possible contribution of 2-AG signaling in the negative consequences of seizure-induced stress-like response cannot be ruled out.

Interestingly, both exposure to stress (Yasmin et al., 2020) and seizure activity (Colangeli et al., 2020) reduced IPSCs which could not be restored following FAAH inhibition. Here we found that metyrapone instead prevented the GABAergic dysfunction induced by seizure activity strongly indicating that both seizures and stress exposure impairs GABA signaling within the BLA through a common mechanism that is independent of AEA signaling. This is in line with the evidence that, unlike excitatory afferents onto BLA pyramidal neurons, GABA terminals are not under a tonic control by AEA signaling (Yasmin et al., 2020; Colangeli et al., 2023). Impaired BLA GABA transmission induced by seizure activity may arise from altered GABA neuron functionality, loss in the number of GABA neurons or both. In the amygdala a significant loss of GABA neurons is consistently found by using animal models of epilepsy that require an initial insult (status epilepticus) to induce the epileptic state (Pitkänen et al., 1998; Aroniadou-Anderjaska et al., 2008). It is generally accepted that standard kindling procedures do not produce obvious neuronal loss - however, extended kindling could result in neuronal loss especially in the hippocampus (Cavazos et al., 1994).

Concerning the effect of kindling on amygdala interneurons, 10 stage-5 seizures have been shown to provoke a significant reduction of GABA neurons in the BLA (Lehmann et al., 1998; Freichel et al., 2004). Conversely, it has been reported that 5 stage-5 seizures yields no reduction in the total number of neurons in the amygdala (Tuunanen et al., 1997) and no decrease in number of GABA-IR amygdala neurons (Callahan et al., 1991). Since our data shows that metyrapone administration prevents the kindling-induced alteration of GABA transmission, this provides some evidence that the repeated seizure-induced CORT surge is responsible for dysregulated GABA activity. In line with our hypothesis it has been reported that repeated CORT administration reduces GABA activity in the amygdala either functionally, by producing a positive shift in the GABAA reversal potential (Duvarci and Paré, 2007) and structurally, reducing GAD67-containing neurons (Lussier et al., 2013). We acknowledge that other mechanisms such as altered chloride homeostasis occur with kindling, and these could also reduce the efficacy of GABA and therefore spike initiation dynamic and overall circuit function that supports behavioural phenotype.

In summary, we elucidated a potential mechanism by which repeated kindled seizures lead to alterations in synaptic function which ultimately manifests as behavioural comorbidities as a potential consequence of AEA downregulation. Our data provides evidence that increases in CORT levels in response to repeated kindled seizures compromises AEA availability within the BLA, a critical modulator for the regulation of emotional processing. Importantly, this study implicates how increased CORT resulting from seizures may benefit from drugs that suppress molecular glucocorticoid signaling. The development of highly selective GR and MR ligands that are at the forefront of clinical use allow a multifaceted approach to treating diseases of aberrant CORT signaling including those with epilepsy.

## CRediT authorship contribution statement

**Renaud C. Gom:** Writing – review & editing, Writing – original draft, Investigation, Data curation, Conceptualization. **Antis G. George:** Writing – review & editing, Formal analysis, Data curation. **Sydney A. Harris:** Writing – review & editing, Data curation. **Pasindu Wickramarachchi:** Writing – review & editing, Data curation. **Dhyey Bhatt:** Writing – review & editing, Data curation. **Shaona Acharjee:** Writing – review & editing, Formal analysis, Data curation. **Quentin J. Pittman:** Writing – review & editing, Supervision. **Matthew N. Hill:** Writing – review & editing, Supervision, Conceptualization. **Roberto Colangeli:** Writing – review & editing, Writing – original draft, Visualization, Formal analysis, Conceptualization. **G. Campbell Teskey:** Writing – review & editing, Supervision, Project administration, Funding acquisition.

## Declaration of competing interest

The authors declare the following financial interests/personal relationships which may be considered as potential competing interests: G. 10.13039/100016655Campbell Teskey reports financial support was provided by 10.13039/100008459University of Calgary. If there are other authors, they declare that they have no known competing financial interests or personal relationships that could have appeared to influence the work reported in this paper.

## Data Availability

Data will be made available on request.
